# Work-related Musculoskeletal Disorders in Korea and Japan: A Comparative Description

**DOI:** 10.1186/2052-4374-26-17

**Published:** 2014-06-24

**Authors:** Eun-A Kim, Minori Nakata

**Affiliations:** 1 Occupational Safety and Health Research Institute, Korea Occupational Safety and Health Agency (KOSHA), 400, Jongga-ro, Jung-gu, Ulsan-city 681-230, Korea; 2 School of Medicine, Department of Social and Environmental Medicine, Kanazawa Medical University, Uchinada, Ishikawa, Japan

**Keywords:** Musculoskeletal disorders, Work-related, Korea, Japan, Comparative review

## Abstract

**Objectives:**

Work related Musculoskeletal disorders (WMSD) is one of the most important problem in occupational health system of Korea and Japan, where the OHS system developed in similar socio-cultural environment. This study compared WMSD in Korea and Japan to review similarities and differences in their historical background, and development of prevention policies.

**Methods:**

Scientific articles, government reports, and related official and non-official statistics on WMSD since the 1960s in Japan and Korea were reviewed.

**Results:**

The historical background and basic structure of the compensation system in Korea and Japan largely overlapped. The issuing of WMSD in both countries appeared as upper limb disorder (ULD), named occupational cervicobrachial diseases (OCD) in Japan, and neck-shoulder-arm syndrome (NSA) 30 years later in Korea, following the change from an industrial structure to automated office work. Both countries developed manuals for diagnosis, guidelines for workplace management, and prevention policies. At present, compensation cases per covered insurers for WMSD are higher in Korea than in Japan, due to the social welfare system and cultural environment. Prevention policies in Korea are enforced more strongly with punitive measures than in Japan. In contrast, the Japanese system requires autonomous effort toward risk control and management, focusing on specific risky processes.

**Conclusions:**

WMSD in Korea and Japan have a similar history of identification and compensation structure, yet different compensation proportions per covered insurer and prevention policies. Follow-up study with international cooperation is necessary to improve both systems.

## Introduction

Musculoskeletal disorders (MSD) have been recognized as a work-related disease since Ramazzini described classical cases in his book from the 18th century [1,2]. After the Industrial Revolution, work-related MSD (WMSD) increased among workers doing repetitive movements, or who were working in a natural standing position for long periods. MSD are currently one of the largest health burdens among humans in modern society. Results from the Global Burden of Disease study project calculated the disease burden worldwide and for 21 regions for 1990, 2005, and 2010, and showed that musculoskeletal disorders (MSD) accounted for 6.8% of total disability-adjusted life years (DALYs), which was much larger than in previous assessments [3].

In terms of specific diseases, low back pain accounted for nearly half, neck pain a fifth, and osteoarthritis about one-tenth of the total MSD. In addition, the proportion of MSD was higher in an aging group compared to a younger group, and higher in Europe, high-income America, and high-income Asia, compared to other regions [3]. According to a European survey carried out in 2005, up to 25% of workers in Europe reported back pain, and 23% of the cases were related to work. Differences between countries are large. For example, in Greece, 47% of workers reported work-related back pain and 46% reported muscular pain, while in the United Kingdom the respective figures were 11% and 9% [4].

In 2002, a Korean burden of disease study showed that DALYs of rheumatoid arthritis were ranked fifth highest DALYs [5]. In addition, 42.2 million (41.2%) Japanese adults reportedly suffer from MSD pain [6]. Thus, as aging societies, Korea and Japan demonstrate similar phenomena regarding increasing MSD proportions among the general population. Because of the high prevalence among the general population, the argument that MSD are work-related is subject to questioning by scientists, governmental agencies, and social opinion groups, regarding clinical definition of the WMSD, criteria of the decision for egronomical exposure, and the work-relatedness of the degenerative MSD. Korea and Japan have shared important experiences regarding occupational health policy and development of management systems, and have shown similar patterns in spite of some differences in their WMSD policy. Korea and Japan shared similar structure of occupational safety and health act and workers’ compensation system; they showed different compensation cases of WMSD, kinds of disorder and the prevention strategy. Understanding past and present of WMSD in these two countries may help to making future direction of the policies for WMSD prevention. This study compared the WMSD issues of Korea and Japan, to review the similarities and differences in their historical backgrounds, development of WMSD policy, and WMSD prevention policy. This comparison could show the triggering of WMSD, compensation system and the main focus of prevention policies in both countries.

## Materials and methods

Scientific articles, government reports, and related official and non-official statistics on WMSD since the 1960s in Japan and Korea were reviewed. Scientific articles were searched through PUBMED, KoreaMed, and Google scholar with the relevant keywords such as musculoskeletal disorder and (Japan or Korea), historical review, key puncher, cervicobrachial diseases, neck shoulder arm syndrome and back pain. Government documents and official statistics were obtained from the homepages of the Ministry of Health, Labour and Welfare of Japan (MLHW) [7], Korea Occupational Safety and Health Agency (,KOSHA) [8] and Ministry of Employment and Labor of Korea (MOEL) [9] The scientific articles described WMSD in Japan since 1960s and in Korea since 1980s were selected. Official statistics of workers’ compensation data of Korea and Japan included not all the injury and disease cases of whole working population.

There are two important notes in understanding Japanese statistics. Firstly, only the cases who were on sick-leave more than four days are counted. Secondly, the official statistics by the Ministry of Health, Labor and Welfare of Japan is not covering the entire workforce of Japan. It includes only the private enterprise workers and self-employed workers. To be precise, Japanese workers are assigned to belong to four different workers’ accident compensation insurance systems depending on employers’ status, namely, accident compensation insurance for private enterprise workers and self-employed workers ( 48 million workers) run by the Ministry of Health, Labor and Welfare, government workers ( 0.3 million workers) run by the National Personnel Authority , local public service workers (3 million workers) run by each local government’s accident compensation fund, and seamen ( 0.1 million workers) by the Ministry of Land, Infrastructure, Transport and Tourism. Similarly Korean workers’ compensation system do not covered the whole working population. It includes private enterprise workers except governmental workers and military workers and private school workers. Compensation cases in Korea counts the cases who were on sick-leave more than four days.

## Results

### Literature survey on Upper limb disorder

Although WMSD were already on the list of occupational diseases in the Japanese and Korean legal systems, they became magnified as a social issue after the introduction of office automation systems. In both countries, WMSD appeared as upper limb disorder (ULD), specifically named occupational cervicobrachial disease (OCD) in Japan, and neck-shoulder-arm syndrome (NSA) 30 years later in Korea. In both countries, the issue arose from office workers whose work was commonly regarded as light work compared with blue collar jobs.

In Japan, tenosynovitis, paratendinitis, and occupational cramps have been found among pianists, telegraphists, and stenographists in the occupational health literature since 1868 [10]. Serious outbreaks of WMSD in Japan followed the introduction of mainframe computer systems in the 1950s, and increasing numbers of key punch operators in banking and stock market businesses around 1960; subsequently, more workers started to suffer from musculoskeletal problems. Because the data processing speeds of computers were so fast, office workers had to enter vast amounts of data rapidly every day. In the early days, this new computer-related illness was known as “key-puncher disease,” as affected workers were engaged exclusively in key-punching work. Most of the sufferers were female [10], and laid claims against the government for relief and acknowledgement of the disorder. However, the Japanese government was reluctant to acknowledge the relationship of work to MSD, as there had been no similar reports worldwide. Even physicians could not yet help sufferers, because the problem was so new.

In the early 1960s, several female workers committed suicide because of long periods of grave physical and mental anguish. Finally, in 1964, the Japanese government acknowledged the illness as a WMSD [11]. This was the world’s first official institutional recognition system for WMSDs derived from computer work. During the 1960s and 1970s, similar illnesses to the “key-puncher disease” were common among various occupations, such as clerks, cashiers, librarians, factory conveyor system line workers, sewing workers, cosmeticians, hairstylists, cooks, dentists, nurses, kindergarten teachers, and professional drivers. In 1972, the Japan Society of Occupational Health (JSOH) gave the name OCD to the WMSD [11]. JSOH recognized OCD as caused by a repetitive, awkward, and excessive workload. Severe cases revealed widespread musculoskeletal pain not limited to the cervicobrachial regions. In addition, severe cases even suffered from depression, sleep disturbance, and autonomic imbalances.

In Korea, during the 1960s–1980s, there were reports of tenosynovitis in athletes [12], and back pain in army women [13], overseas workers [14], and miners [15]. In the Korean legal system, WMSD has been defined as part of the schedule of occupational diseases since the enforcement of the labor standard act (EDLSA) in 1954 [16]. In the EDLSA, the WMSD is described as “diseases of muscles, tendons, and articulates due to heavy work; neuritis or other disorders from using a drill or rivet gun; or finger tremors or writers’ spasms in telegraphers, typists, and scribes [13]”. However, most cases involving the musculoskeletal system up until the late 1980s were back pain after occupational accidents, which were classified as occupational injuries, not diseases [17].

After the media reported on the first law in the United States regulating video display terminal (VDT) use in the workplace in 1988 in Suffolk County, New York, [18], non-governmental organizations began surveys to look at occupational diseases in possible industries. The first major outbreaks of ULD in Korea appeared in telephone operators. Since the introduction of the telephone in 1882, users increased to 10 million in 1987. In the late 1980s, operators in a large telephone company were investigated for continuous pain of the neck, shoulders, and arms [19-23]. A comprehensive survey of the telephone company employees revealed that the operators posture during work was ergonomically inappropriate, causing the workers’ WMSD [21]. According to the study, the most frequent symptom of the telephone operators was muscle tenderness in their shoulders and neck [21]; a muscle tenderness examination and the Morley test were recommended for accurate diagnosis [20]. In 1994, NSA was listed on the EDLSA as a compensable work-related disorder, and 20 cases of work-related NSA were found that year [17,24]. Several surveys for NSA were conducted by NGOs and participating academics [25], and social concern regarding the health effects of VDT use in the workplace increased. In 1997, the Ministry of Labor stipulated guidelines for VDT workers (Ministry of Labor No. 1997–8), including recommendations for ergonomics, posture, management of working schedule and rest, and working environment.

Another important issue regarding WMSD in Korea was the increase in applications for workers’ compensation, especially after 2000 [26,27]. The most important factor in the increase was the Union’s activity, especially in shipbuilding and car manufacturing industries, to promote WMSD compensation. From 2002–2004, 21% of all WMSD compensation cases were workers from 11 large companies. Most of the compensated cases were for low back pain, including acute injuries and chronic illness [26].

### WMSD compensation statistics

Compensation for WMSD over the past 9 years (Table 1) was greater in Japan up until 2005, and has declined since 2006. The increase of WMSD in Korea resulted from the work-related disorder classification system that was introduced in 2006, when low back pain by accident was included as a work-related disease that was formerly classified to injury [26]. After the highest counts in 2007, WMSD subsequently decreased in Korea. The frequency of compensated WMSD in both countries has been approximately 5,000 cases each year.

**Table 1:** Specific types of WMSD compensation in Korea and Japan

**Year**	**2004**	**2005**	**2006**	**2007**	**2008**	**2009**	**2010**	**2011**	**2012**
Korea									
ALBP	-	-	3,612	5,769	3,401	2,472	1,720	2,556	2,779
NALBP	-	975	1,006	564	1,831	2,407	2,288	1,168	1,013
Over-exertion	2,953	1,926	1,615	1,390	1,471	1343	1,292	1,161	1,438
CTS	-	-		-	30	12	202	192	97
Vibration	2	16	9	16	7	9	15	23	19
WMSD	2,955	2,917	6,242	7,739	6,740	6,243	5,517	5,100	5,346
Japan									
ALBP	4,377	4,840	4,889	5,230	5,509	4,816	4,960	4,766	4,789
NALBP	54	55	31	57	47	54	58	56	43
Over-exertion	89	105	92	119	89	109	117	87	90
Finger, forearm	154	180	233	245	246	163	141	161	139
Vibration	9	4	6	5	3	3	5	4	9
Other	62	81	70	92	105	59	73	73	91
WMSD	4,745	5,265	5,321	5,748	5,999	5,204	5,354	5,147	5,161

- : not available due to the classification system, ALBP: accident-related low back pain, NALBP: non-accidental low back pain, Over-exertion: other musculoskeletal disorder due to over exertion, CTS: carpal tunnel syndrome, WMSD: work-related musculoskeletal disorder.

Differences in compensated WMSD between Japan and Korea are presented for specific diseases (Table 1), and for covered workers (Table 2). The ratio of low back pain caused by accidents versus non-accidental low back pain is extremely different in the two countries. In addition, despite similar overall low back pain counts, the relative counts per covered insurer are higher in Korea than Japan (Table 2). The WMSD compensation counts per 10^5^ covered workers are 22.5–61.8 in Korea, and 9.7–11.4 in Japan. Not only WMSD, but also total injury and disease, and all disease counts per covered workers are also higher in Korea than in Japan. In 2011, 73% of Japanese expenditures for health came from social security, and 15% came from private individuals. The same percentages for Korea were 46% and 37% respectively (Table 3) [28].

**Table 2:** WMSD compensation frequencies for workers covered by insurance in Korea and Japan

**Year**	**2004**	**2005**	**2006**	**2007**	**2008**	**2009**	**2010**	**2011**	**2012**
**Korea**									
Covered workers	10,473,090	11,059,193	11,688,797	12,528,879	13,489,986	13,884,927	14,198,748	14,362,372	15,548,423
Total injury & disease	88,874	85,411	89,910	90,147	95,806	97,821	98,645	93,292	92,256
Disease	9,183	7,495	10,235	11,472	9,734	8721	7,803	7,247	7,472
WMSD	2,955	2,917	2,630	7,739	6,740	6,243	5,517	5,100	5,346
Compensation cases/10^5^ covered workers									
WMSD	28.2	26.4	22.5	61.8	50.0	45.0	38.9	35.5	34.4
Disease	87.7	67.8	87.6	91.6	72.2	62.8	55.0	50.5	48.1
Total injury & disease	848.6	772.3	769.2	719.5	710.2	704.5	694.7	649.6	593.3
**Japan**									
Covered workers	48,552,436	49,184,518	50,707,376	51,313,223	52,418,376	52,788,681	52,487,983	52,741,870	53,236,873
Total injury & disease	122,804	120,354	121,378	121,356	119,291	105,718	107,759	117,958	119,576
Disease	7,609	8,226	8,369	8,634	8,874	7,491	8,111	7,779	7,743
WMSD	4,745	5,265	5,321	5,748	5,999	5,204	5,354	5,147	5,161
Compensation cases/10^5^ covered workers									
WMSD	9.8	10.7	10.5	11.2	11.4	9.9	10.2	9.8	9.7
Disease	15.7	16.7	16.5	16.8	16.9	14.2	15.5	14.7	14.5
Total injury & disease	252.9	244.7	239.4	236.5	227.6	200.3	205.3	223.7	224.6

WMSD: work-related musculoskeletal disorder.

**Table 3:** Health care system in social security of Korea and Japan

	**Korea**	**Japan**
Expenditures on health by type of funding (%)		
GG/SS/POP/PI	11/46/37/6	9/73/15/2
Share of hospital inpatient expenditures for MSD (%)	9	6
Health care system		
Out-of-pocket healthcare expenditures as a share of total household consumption (%)	4.6	2.2
Sickness leave	No	66.7% of average daily basic wage up to 18 months
Share of WMSD in workers’ compensation (%)		
WMSD/total compensated diseases	71.5	66.6
WMSD/total compensated injury and diseases	5.8	4.3

GS: general governmental, SS: social security, POP: private out-of-pocket, PI: private insurance, MSD: musculoskeletal disorder, WMSD: work-related musculoskeletal disorder.

Since 2010, despite similar frequencies of occurrence, the specific types of WMSD were different in Korea and Japan. The first difference was the composition of low back pain. Most of the compensated cases of low back pain in Japan were accident-related (99%). In Korea, the average proportion of accident-related low back pain cases was 67% from 2006–2012. The second difference related to “other musculoskeletal disorders from over exertion (labelled ‘over exertion’)” which include the neck, shoulder, and upper arm region. This category has been continuously decreasing in Korea, but is still 16 times higher than in Japan (Table 1). WMSD in Japan include the category “finger, forearm”, which might relate to carpal tunnel syndrome (CTS) in Korea. WMSD of the finger and forearm region (with the exception of CTS) is included in the “over exertion” category in Korea. Finally, vibration-related WMSD cases were higher in Korea than in Japan.

### WMSD prevention policies

In Korea Employers’ duties regarding computerized systems in the workplace were described briefly in a ministerial decree of occupational health standards in 1990 [29]. With increasing WMSD compensation cases and social concern, the Korean MOL prepared best practices for WMSD prevention in 2000, based on practices in other countries. From 2002 onward, employers were obliged to take necessary measures for the prevention of simple and repetitive work, or excessive physical labor, according to Article 24 (Health Measure) of the Korean occupational safety and health act [29]. Based on a ministerial decree (2003) [29], employers’ duties for WMSD prevention included: risk assessment, definition of high-risk WMSD, improvement of the workplace, workers’ right-to-know, medical support for WMSD workers, and planning a WMSD prevention program. Specific guidelines for heavy material were also stipulated. This enforcement included a penalty clause, which carries the strongest fine among the clauses of the Occupational Health and Safety Act in Korea (Table 4).

**Table 4:** WMSD prevention policies in Korea and Japan

	**Korea**	**Japan**
Evaluation of risk factors	Health and Safety act requires the employer to evaluate risk factors for WMSD	No obligation
High-risk jobs	Eleven kinds of jobs at high risk for WMSD as specified by MOEL	No list
WMSD prevention program	Employer should put into place a WMSD prevention program according to the enforcement regulations when there is a high risk for WMSD	Established regulations for prevention of low back pain, OCD, and vibration disorders
Medical examination	Does not exist	Recommended
Penalty	Violation penalty	Does not exist
Other	Employer obliged to improve the workplace and make workers aware of the risk	Counseling service at the regional occupational health center for WMSD prevention

WMSD: work-related musculoskeletal disorder, OCD: occupational cervicobrachial disease, MOEL: Ministry of Employment and Labor.

The Japanese system for WMSD prevention is based on recommendations to the employer. Most of the recommendations from the Ministry of Labor are for low back pain, OCD, and vibration disorder. Five notifications for OCD have been specified since 1964: medical examinations for key-puncher work (Ministry of Labor, no 1106, 1964), medical examinations for cash register work (Ministry of Labor, no 188 and 717, 1973), prevention of finger diseases for tools with triggers (Ministry of Labor, no 94, 1975), and an occupational health guide and medical examination for VDT work (Ministry of Labor, no 705, 1985). In terms of low back pain prevention, a “guideline for workplace low back pain” was specified in 1994 (Ministry of Labor, no 547). Two guidelines for vibration disorders have been specified since 1975. All of the notifications are simply recommendations for the employer, without penalty clauses (Table 4).

## Discussion

WMSD in Korea and Japan have received attention with increases in cases of neck, shoulder, and upper limb disorders. Although the crucial risk factors for ULD in the workplace are repetitive work and fixed posture, which commonly occur in various manufacturing industries, the triggering job for recognition of ULDs in both countries was clerical work, which is generally sedentary and not commonly associated with heavy physical workloads [30]. The Japanese experience with ULDs started in the 1960s with the popularization of the keyboard and video display unit [10]; this was earlier than VDT syndrome seen in Australia in the mid-1980s and in the United States in the 1990s [30]. In the 1980s, the VDT and keyboard also became common in Korean society. After 30 years the Korean experience was also largely initiated through the involvement of telephone operators. A relatively large telephone company, with support from the labor union and NGOs, launched an effort to obtain occupational compensation. Abrupt increases in WMSD compensation cases in Korea were the result of those grouped requests for compensation from large companies. Thus, both Japan and Korea experienced similar patterns of emergence regarding ULDs and WMSD in clerical workers.

In 1971, the Japan Association of Industrial Health (JAIH) organized a committee on cervicobrachial syndrome, through which a definition of OCD, diagnostic criteria, and a health examination manual and checklists were developed [11]. In the definition developed by the JAIH committee, mental symptoms, such as emotional instability, difficulty concentrating, sleep disturbances, depressive state, and hysterical state, were not ignored. OCD is defined by JSOH as a disorder of occupational origin, involving functional and/or organic disturbances resulting from neuromuscular fatigue, due to working in a fixed position or with repetitive movements of the upper extremities. In contrast, the mental and psychological aspects of ULD in repetitive sedentary work have not been considered in Korea. The identification of NSA in Korea depends on medical findings, and specifically the neurological examination and ergonomic evaluation, without concern for psychological and mental aspects. Therefore, Japan and Korea show differences in the definitions of OCD and NSA with respect to the inclusion of psychological functioning.

Korea and Japan have shared a similar strategy for occupational WMSD compensation through workers’ insurance systems. On the other hand, in terms of prevention policy, there are differences between the two countries. The Korean system operated on the stronger enforcement of the employer focusing on risk assessment for WMSD, including violation penalty. In contrast to the Korean system, Japanese policy requires the employer to engage in autonomous prevention activity, based on specific guidelines provided through notifications from the Ministry of Labor. No penalties are stipulated in these notifications (Table 4). As a leader regarding ULDs in visual display terminal (VDT) workers, Japanese research achievements with respect to diagnostic criteria and pathophysiology have aided the development of the Korean system, beyond diagnosis and pathology, toward risk assessment and control as a result.

Japan and Korea showed differences in the ratios of accidental versus non-accidental low back pain. These differences might be the result of different concepts of accidents and diseases in the two countries. Total counts of low back pain have been similar in both countries in recent years, and the proportion of WMSD to total compensated disease in both countries is similar (71.5% in Korea and 66.6% in Japan). Therefore, the composition of WMSD compared to other diseases in the compensation system in both Korea and Japan seemed to be similar.

Overall, occurrences for insured workers of WMSD, injury, and disease were higher in Korea than in Japan. One possible explanation for this is the difference between the social welfare systems of the two countries. Because expenditures for MSD hospital inpatients have been higher in Korea (9%) than Japan (6%), MSD have been a more pressing burden for Korean society. Therefore, workers need more healthcare support from social security either through health insurance or through workers’ insurance. Importantly, the national health care system in Korea does not offer a paid sickness leave plan, which is included in a workers’ compensation system. For this reason, workers tend to seek workers’ compensation eagerly. Another possible explanation relates to differences in the social or cultural environments in two counties, which encourage or discourage workers from obtaining compensation.

Although the Industrial Safety and Health Law in Japan and Occupational safety and Health Act in Korea is applied in common to all workers, accident compensation insurance system of both countries were not necessarily applying common rules for acceptance of work-related accidents and occupational diseases. Each accident compensation insurance system reports statistics in its own manner applying somewhat different classification systems. Therefore, it is not possible to know exact overall figures of workers’ occupational accidents and occupational diseases in both countries which is one of the limitations of this study. However, the similar composition of workers’ compensation scheme on two countries, gross comparison between two countries was possible. Another limitation of this study is lack of the analysis the effectiveness of the prevention strategy of two countries. To compare the effectiveness of the prevention strategy need further study on comprehensive socioeconomic review and cost benefit analysis.

## Conclusion

The present study compared and contrasted Japan and Korea in terms of WMSD origins, compensation, and prevention policies. Because WMSD in Korea and Japan have shared a similar historical trajectory, many prevention and compensation policies have overlapped. Comparing the beginning and the present status of WMSD in Korea and Japan, WMSD of both countries increased with rises in ULD (such as OCD in Japan and NSS in Korea), especially among clerical workers. In both countries, the change from an industrial structure to automated office work seems to have spurred this increase. Both countries have developed manuals for diagnosis, guidelines for workplace management, and prevention policies. Presently, compensation cases per covered insurers for WMSD are higher in Korea than Japan, due to social welfare and the cultural environment, but the composition of WMSD among work-related diseases is similar. Prevention policies in Korea involve stricter enforcement with respect to employers than in Japan. The Japanese system requires autonomous effort toward risk control and management, focusing on specific risky processes. In order to improve both systems, follow up study with international cooperation is necessary.

## Abbreviations

WMSD: Work-related musculoskeletal disorders; ULD: Upper limb disorders; OCD: Occupational cervicobrachial diseases; NSA: Neck-shoulder-arm syndrome; MSD: Musculoskeletal disorders; DALYs: Disability-adjusted life years; VDT: Video display terminal.

## Competing interests

The authors disclose have no conflicts of interests.

## Authors’ contributions

EAK planned the study, structured the statement, review and analysis of the data, and constructed description for Korean system. MN reviewed and analysis the Japanese system, statistics and the review all the draft and revise. The results, discussion and the conclusion were reviewed both authors. Both authors read and approved the final manuscript.
